# Mating type specific chromosome conformation in *Saccharomyces cerevisiae*

**DOI:** 10.1186/1756-8935-6-S1-P92

**Published:** 2013-03-18

**Authors:** Jon-Matthew Belton, Davide Bau, Imen Lassardi, Kerstin Bystricky, Marc Marti-Renom, Job Dekker

**Affiliations:** 1 Program in Systems Biology, Program in Gene Function and Expression, Department of Biochemistry and Molecular Pharmacology, University of Massachusetts Medical School, Worcester MA 01605, USA; 2 Structural Genomics Team, CNAG - Centro Nacional de Análisis Genómico and Structural Genomics Group - Gene Regulation, Stem Cells and Cancer Program - Center for Genomic Regulation (CRG), Barcelona Spain; 3 LBME - CNRS University of Toulouse, France

## 

Budding yeast switch their mating type by a gene conversion event at the *MAT* locus which uses either of two silent loci (*HML* or *HMR*) on opposite ends of chromosome three as a template. In *MATa* cells the left arm of Chr. Ill is “activated” which allows for the preferential recombination of *HML* with the *MAT* locus. The left arm is otherwise “repressed” for recombination in *MATα* cells which then prefer to use *HMR*, on the right arm, as a template for gene conversion. We set out to analyze the potential role of chromosome conformation in this “activation”/”repression” phenomenon observed on the left arm of Chr. III. We used Chromosome Conformation Capture Carbon Copy (5C) to comprehensively analyze the conformation of chromosomes III, V, and XII in the two mating types. Our data reveals that the yeast genome is organized in a unique way compared to other species. We have found that global nuclearorganization such ascentromereclustering, telomere tethering to the periphery, and sequestration of the rDNA array into the nucleolus affect both the specific conformations of each chromosome but also the interactions between these chromosomes. Our analysis indicates that the overall architecture for these 3 chromosomes is very similar between the two mating types. Interestingly, a mating type specific difference in conformation of the left arm of Chr. Ill was identified. Furthermore, the 5C data was used, in conjunction with the Integrative Modeling Platform (IMP), to generate three dimensional models of Chr. III in both mating types. This method provides a more intuitive way of viewing 5C data and reveals that, in general, Chr. Ill has a more crumpled conformation in *MAT**a*** cells than in *MAT**α**.* However, this crumpling is most evident on the left arm of the chromosome. Thus the phenomenon of “activation”/”repression” of the left arm of Chr. III which is associated with mating type-specific switching preference is, in fact, associated with a difference in the innate conformation of Chr. Ill between the two mating types. This difference in structure between mating types will be used as a phenotype to analyze the effect of cis and trans acting factors that play a role in switching preference through alteration of chromosome conformation.

